# Value of total tumor load as a clinical and pathological factor in the prognosis of breast cancer patients receiving neoadjuvant treatment. Comparison of three populations with three different surgical approaches: NEOVATTL Pro 3 Study

**DOI:** 10.1007/s10549-023-06954-8

**Published:** 2023-05-23

**Authors:** María Dolores Martín-Salvago, Magdalena Sancho, M. Ángeles López-García, Alicia Cano Jiménez, Ana Pérez-Luque, Lina Alfaro, Begoña Vieites

**Affiliations:** 1 Department of Pathology, Hospital Universitario Virgen del Rocío, Seville, Spain; 2 Department of Pathology, Hospital Universitario de Jaén, Jaén, Spain; 3 Department of Pathology, Complejo Asistencial Universitario de Salamanca, Salamanca, Spain; 4 Department of Gynaecology and Obstetrics, Hospital Universitario Virgen del Rocío, Seville, Spain; 5 Medical Oncology Department. Hospital, Universitario de Jaén, Jaén, Spain

**Keywords:** Breast cancer, Axillary lymphadenectomy, Total tumor load, Sentinel lymph node biopsy, Neoadjuvant systemic treatment

## Abstract

**Purpose:**

This study aimed to compare the prognosis in terms of disease-free survival (DFS) in three populations of women with breast cancer (BC) treated with neoadjuvant systemic treatment (NAST) in which axillary lymph node dissection (ALND) was performed based on different total tumor load (TTL) thresholds in the sentinel nodes.

**Methods:**

This was an observational, retrospective study carried out in three Spanish centers. Data from patients with infiltrating BC who underwent BC surgery after NAST and intraoperative sentinel lymph node biopsy (SLNB) performed by One Step Nucleic acid Amplification (OSNA) technique during 2017 and 2018 were analyzed. ALND was performed according to the protocol of each center, based on three different TTL cut-offs (TTL > 250, TTL > 5000, and TTL > 15,000 CK19-mRNA copies/μL for centers 1, 2, and 3, respectively).

**Results:**

A total of 157 BC patients were included in the study. No significant differences in DFS were observed between centers (Hazard ratio [HR] center 2 vs 1: 0.77; *p* = 0.707; HR center 3 vs 1: 0.83; *p* = 0.799). Patients with ALND had a shorter DFS (HR 2.43; *p* = 0.136), albeit not statistically significant. Patients with a triple negative subtype had a worse prognosis than those with other molecular subtypes (HR 2.82; *p* = 0.056).

**Conclusion:**

No significant differences in DFS were observed between three centers with different surgical approaches to ALND based on different TTL cut-offs in patients with BC after NAST. These results suggest that restricting ALND to those patients with TTL ≥ 15,000 copies/μL is a reliable approximation, avoiding unnecessary morbidities caused by ALND.

**Supplementary Information:**

The online version contains supplementary material available at 10.1007/s10549-023-06954-8.

## Introduction

Breast cancer (BC) is one of the main life-threatening neoplasms in women worldwide [1]. In Spain, BC is the leading cause of cancer and cancer-related death in women [2]. The use of neoadjuvant systemic treatment (NAST) as a preoperative treatment modality is currently being extended to earlier stages as it offers a wide range of benefits, including the opportunity to further de-escalate the surgical management of the axilla [3–6]. However, there is still a lack of consensus regarding the post-treatment surgical approach to axillary lymph node dissection (ALND).

Contrary to ALND, sentinel lymph node biopsy (SLNB) is a minimally invasive procedure that allows accurate axillary nodal staging with less morbidity. It has been established as the gold standard for pathological evaluation of the axilla in patients with operable BC and clinically negative axilla. SLNB diagnostic performance by conventional histopathology following NAST remains controversial, since this treatment may affect the lymphatic drainage and causes tissue alterations, limiting the nodal histological evaluation [7–9]. Hence, the use of a molecular method may be more advisable.

One Step Nucleic acid Amplification (OSNA) is a molecular technique that allows the detection of the number of copies of messenger Ribonucleic Acid (mRNA) of cytokeratin 19 (CK19) present in the sentinel lymph node (SLN). CK19 is a membrane protein expressed by most breast carcinomas even after NAST, constituting a good target to detect BC metastasis [10, 11].The calculation of the total tumor load (TTL) based on CK19 mRNA copy number is a highly sensitive and specific method for the detection of micro and macrometastasis of lymph nodes (LNs) [12, 13]. TTL is a quantitative index that provides information on the metastatic load present in the LNs, in an objective manner. Furthermore, the OSNA method is an automated and reproducible approach to calculate TTL using metastatic load information [14]. Therefore, its use could provide more sensitive information regarding the response to NAST in the LNs compared with conventional histological examination, limiting overtreatment or unnecessary ALND and adding more precision to the axillary surgical management [7, 15].

The prognostic value of TTL, measured by OSNA, was previously demonstrated in a population of BC patients that did not receive NAST (PLUTTO study), confirming the clinical value of the TTL. Furthermore, this study established a correlation of TTL with disease-free survival (DFS) and defined two risk groups (TTL < 25,000 copies/μL: low-risk and TTL ≥ 25,000 copies/μL: high-risk) [16]. This prognostic value of TTL, measured using the OSNA technique, was also demonstrated for BC patients after NAST in the NEOVATTL study [17], showing a clear decrease in survival at TTL ≥ 25,000 copies/μL. Although this prognostic value of TTL seems to be valid in the population with and without NAST, the subsequent surgical strategy to the axillary approach, based on the results of the SLN assay and TTL values, varies widely in clinical practice. In this regard, clinical guidelines recommend performing ALND in presence of any nodal involvement (even isolated-tumor cells and/or micrometastases) [18]. Moreover, the NEOVATTL study showed that TTL values > 15,000 copies/μL were predictive of LN involvement after intraoperative OSNA assay in patients with BC after NAST.

Despite the predictive and prognostic value of TTL derived from intra-operative OSNA assay in SLN of BC patients after NAST was demonstrated in the NEOVATTL study, evidence is still scarce. These data could be relevant to develop protocols using TTL cut-off points to guide the surgical strategy regarding the axillary approach in these patients. This retrospective study aimed to compare the prognosis in terms of disease-free survival (DFS), in three populations of women with BC treated with NAST in which TTL values were used as criteria to perform ALND.

## Materials and methods

### Study design and population

This was an observational, retrospective, multicenter study of clinicopathologic data registry analysis and follow-up of women with infiltrating BC who had undergone BC surgery and SLNB. Patients with at least 3 years of follow-up who received NAST followed by SLNB with OSNA technique during 2017 and 2018 were included in the study. Patients under 18 years of age, with carcinoma in situ or other malignant neoplasms, and those considered unsuitable for the study by the investigator were excluded, such as those with no migration of the tracer used in the SLNB during surgery, precluding detection of the SLN, and those lost to follow-up.

This study was carried out in three Spanish centers. Surgeons performed ALND according to the protocol of each center, based on three different TTL cut-offs. Thus, patients with TTL > 250 (center 1), TTL > 5000 (center 2), TTL > 15,000 (center 3) CK19 mRNA copies/μL were selected for ALND in each center. Data from those patients meeting the eligibility criteria of the study were collected from the digital medical records in September–October 2021. The investigator included each patient’s data in the case report form in an anonymized manner.

This study was performed after approval from the Comité Ético provincial de Andalucía, PEIBA (Ethics Committee of Andalucía) and the Instituto de Investigación Biomédica de Salamanca (IBSAL) (Salamanca Biomedical Research Institute). Furthermore, it was developed following the ethical principles originating from the latest version of the Declaration of Helsinki accepted by local authorities and which are in line with Good Clinical Practice (GCP) and the requirements of current Spanish regulations.

### Endpoints, variables, and assessments

The main objective of this study was to compare the prognosis in terms of DFS in three populations of women with BC treated with NAST (considering DFS as the time until evidence of disease recurrence or progression was found). The three populations differed in the criteria for the indication for ALND, which was TTL > 250, TTL > 5000, and TTL > 15,000 CK19 mRNA copies/μL (measured using the OSNA technique) according to each center's protocol. The secondary objectives were to compare the prognosis after NAST in the three populations (i.e., with different criteria for ALND indication) according to tumor subtype and to calculate the percentage of unnecessary ALND in the three populations, as the weighted mean of ALND with no positive LNs at each center.

In addition to variables associated with the primary objective (i.e., tumor recurrence location and date) and demographic characteristics (i.e., age), previous tumor features and type of neoadjuvant, surgical, and adjuvant treatments were collected. Tumor characteristics included TNM classification, histological type, grade, hormonal receptors, HER2 status, Ki67 (% of positive cells), and molecular subtype. SLNB analysis before neoadjuvant treatment (yes/no), type of neoadjuvant treatment (chemotherapy/hormonotherapy), and radiological evaluation of response to NAST (in breast and axilla) were also recorded. Surgery characteristics evaluated were the type of breast surgery (conservative/radical), data related to SLNB (OSNA evaluation, tumor load per node analyzed, and TTL), and ALND (yes/no; non-sentinel metastatic nodes/non-sentinel nodes removed). Adjuvant strategies were additionally collected, including type of treatment: chemotherapy (yes/no), hormone (yes/no), and radiotherapy (yes/no). Finally, recurrence disease (site and date) and exitus (yes/no; date) was also considered.

### Statistical analysis

A descriptive analysis was performed for all variables of interest using centralization and dispersion parameters. Quantitative variables were described using the mean and the standard deviation (SD), and the median (P50) together with the 25th and 75th percentiles (P25–P75) as well as the minimum and maximum values. Qualitative variables were described using the absolute frequencies and percentages together with the 95% confidence interval (95% CI).

To achieve the main objective, the DFS of the sample was described using the Kaplan–Meier analysis, considering the recurrence or progression of the disease as an event. Furthermore, Kaplan–Meier curves were compared between different risk groups. Differences between these groups were evaluated by hazard ratio (HR) (95% CI). Statistical significance (*p* value) was computed using log-rank test.

For the other objectives, univariate Cox regression analyses were performed to estimate the HR of recurrence/progression associated with each of the potential predictive variables of interest, including TTL cut-off for ALND in each center, ALND, and molecular subtype (independent variables) and time to event as the dependent variable. Center 1 and no ALND were used as references for the calculation of the HRs when comparisons were made between centers and ALND (yes/no).

All analyses were conducted with the R language (version 4.2.1) installed on windows 10, using functions implemented in the Survival and survminer packages for Cox model fitting and evaluation.

## Results

### Characteristics of the study population

A total of 157 BC patients were included in the study, of which 28 belonged to center 1, 72 to center 2, and 57 to center 3. The mean age of the patients was 59 (±SD 13.1) years and was similar between the different centers. Most of the patients had a cT2N0 and were diagnosed with invasive breast carcinoma, not otherwise specified (NOS) histologic type (Table 1). The hormonal receptors and HER2 status, the molecular subtype and data related to neoadjuvant treatment are included in Tables S1 and S2 (found in Online Resource 1). There were no differences in TTL values according to molecular subtype (other vs. triple negative).

**Table 1:** Previous patient characteristics according to center

	Total	C1	C2	C3
Age (years—day of surgery)				
N	157	28	72	57
Mean (SD)	59.2 (13.1)	60.2 (11.7)	59.5 (12.3)	58.2 (14.7)
* P*-value^(1)^				
C1		–	0.7957	0.5056
C2		0.7957	–	0.5983
C3		0.5056	0.5983	–
cT, *n* (%)				
N	156	28	72	56
cT1	20 (12.8)	0 (0)	9 (12.5)	11 (19.6)
cT2	113 (72.4)	25 (89.3)	51 (70.8)	37 (66.1)
cT3	22 (14.1)	3 (10.7)	12 (16.7)	7 (12.5)
cT4	1 (0.6)	0 (0)	0 (0)	1 (1.8)
* P*-value^(2)^				
C1		–	0.5474	0.2606
C2		0.5474	–	0.4554
C3		0.2606	0.4554	–
cN, *n* (%)				
N	156	28	72	56
cN0	117 (75.0)	21 (75.0)	58 (80.6)	38 (67.9)
cN1	39 (25.0)	7 (25.0)	14 (19.4)	18 (32.1)
* P*-value^(3)^				
C1		–	0.5887	0.6156
C2		0.5887	–	0.1060
C3		0.6156	0.1060	–
Histological subtype, *n* (%)				
N	157	28	72	57
NOS	91 (58.0)	27 (96.4)	64 (88.9)	0 (0)
Ca NOS	46 (29.3)	0 (0)	0 (0)	46 (80.7)
Other	11 (7.0)	1 (3.6)	3 (4.2)	7 (12.3)
ILC	9 (5.7)	0 (0)	5 (6.9)	4 (7.0)
* P*-value^(4)^				
C1		–	0.3520	< 0.0001
C2		0.3520	–	< 0.0001
C3		< 0.0001	< 0.0001	–
Grade, *n* (%)				
N	155	28	71	56
G1	15 (9.7)	1 (3.6%)	10 (14.1%)	4 (7.1%)
G2	77 (49.7)	9 (32.1)	35 (49.3)	33 (58.9)
G3	63 (40.6)	18 (64.3)	26 (36.6)	19 (33.9)
* P*-value^(2)^				
C1		–	0.0107	0.0144
C2		0.0107	–	0.7097
C3		0.0144	0.7097	–

*C1* center 1, *C2* center 2, *C3* center 3, *SD* standard deviation, *cT* clinical tumor stage, *cN* clinical regional lymph node stage, *NOS* invasive breast carcinoma, not otherwise specified, *Ca NOS* carcinoma not otherwise specified, *ILC* invasive lobular carcinoma

^(1)^*t* test independent data comparing each center with the other two

^(2)^Linear-by-linear association test comparing each center with the other two

^(3)^Fisher’s exact test comparing each center with the other two

^(4)^Pearson chi-squared test comparing each center with the other two

### Surgery characteristics and TTL

Regarding the characteristics of the surgery, most patients underwent a conservative surgical approach. A total of 136 patients did not undergo ALND. The remaining 21 patients underwent ALND (7 from center 1, 8 from center 2, and 6 from center 3). Most patients (belonging to centers 2 and 3) lacked metastatic non-sentinel node compromise. In centers 1 and 2, 14.3% and 25% of the 7 and 8 patients with ALND, respectively, presented negative lymphadenectomy, which could have been avoided by applying the 15,000 copies cut-off criterium (Table S4 found in Online Resource 1). Based on the primary outcome, the percentage of unnecessary ALND, defined as negative ALND in patients with < 15,000 copies/μL, in the three centers was 14.3% (Table 2).

**Table 2:** Surgery characteristics according to center

	Total	C1	C2	C3
Surgery type, *n* (%)				
N	157	28	72	57
Conservative	110 (70.1)	24 (85.7)	46 (63.9)	40 (70.2)
Radical	47 (29.9)	4 (14.3)	26 (36.1)	17 (29.8)
* P*-value^(1)^				
C1		–	0.0503	0.1806
C2		0.0503	–	0.5729
C3		0.1806	0.5729	–
TTL (copies/μL)				
N	157	28	72	57
Mean (SD)	25,784 (136,942)	4,170 (13,371)	35,813 (179,530)	23,733 (104,512)
* P*-value^(2)^				
C1^*^		–	0.9128	0.6673
C2^*^		0.9128	–	0.6967
C3^*^		0.6673	0.6967	–
Log_10_ TTL (copies/μL)				
N	157	28	72	57
Mean (SD)	1.0 (1.7)	0.9 (1.7)	1.0 (1.7)	1.1 (1.8)
* P*-value^(2)^				
C1		–	0.9128	0.6673
C2		0.9128	–	0.6967
C3		0.6673	0.6967	–
TTL (copies/μL), *n* (%)				
N	157	28	72	57
< 250	114 (72.6)	21 (75.0)	53 (73.6)	40 (70.2)
> 250 to 5000	23 (14.6)	2 (7.1)	11 (15.)	10 (17.5)
> 5000 to 15,000	7 (4.5)	3 (10.7)	3 (4.2)	1 (1.8)
> 15,000	13 (8.3)	2 (7.1)	5 (6.9)	6 (10.5)
* P*-value^(3)^				
C1		–	0.7798	0.9053
C2		0.7798	–	0.6122
C3		0.9053	0.6122	–
OSNA, *n* (%)				
N	157	28	72	57
No	2 (1.3)	0 (0)	0 (0)	2 (3.5)
Yes	155 (98.7)	28 (100)	72 (100)	55 (96.5)
* P*-value^(1)^				
C1		–	1.0000	1.0000
C2		1.0000	–	0.1933
C3		1.0000	0.1933	–
ALND, *n* (%)				
N	157	28	72	57
No	136 (86.6)	21 (75.0)	64 (88.9)	51 (89.5)
Yes	21 (13.4)	7 (25.0)	8 (11.1)	6 (10.5)
* P*-value^(1)^				
C1		–	0.1167	0.1102
C2		0.1167	–	1.0000
C3		0.1102	1.000	–
No metastatic non-sentinel node, *n* (%)				
N	157	28	72	57
0	146 (93.0)	22 (78.6)	68 (94.4)	56 (98.2)
1	2 (1.3)	0 (0)	2 (2.8)	0 (0)
2	1 (0.6)	0 (0)	0 (0)	1 (1.8)
3	2 (1.3)	2 (7.1)	0 (0)	0 (0)
4	1 (0.6)	0 (0)	1 (1.4)	0 (0)
5	1 (0.6)	0 (0)	1 (1.4)	0 (0)
6	1 (0.6)	1 (3.6)	0 (0)	0 (0)
9	1 (0.6)	1 (3.6)	0 (0)	0 (0)
11	2 (1.3)	2 (7.1)	0 (0)	0 (0)
* P*-value^(3)^				
C1		–	< 0.0001	0.0212
C2		< 0.0001	–	0.1446
C3		0.0212	0.144	–

*C1* center 1, *C2* center 2, *C3* center 3, *SD* standard deviation, *TTL* total tumor load, *OSNA* One Step Nucleic acid Amplification, *ALND* axillary lymph node dissection

^(1)^Fisher’s exact test comparing each center with the other two

^(2)^Mann–Whitney test comparing each center with the other two

^(3)^Linear-by-linear association test comparing each center with the other two

### DFS according to center and ALND

A total of 14 patients relapsed, with a similar distribution between centers and without a predominant body location. Additionally, seven patients died (Table 3). No significant differences were found in DFS according to the type of surgery (conservative or radical) (HR 1.30 95% CI 0.43–3.87; *p* = 0.642). Regarding TTL values of non-relapsing patients, 74.8% had a TTL value under 250 copies/μL, and 7.0% had a TTL value above 15,000 copies/μL (Table 4).

**Table 3:** Tumor recurrence and exitus according to center, n (%)

	Total	C1	C2	C3
Recurrence				
N	157	28	72	57
No	143 (91.1)	25 (89.3)	66 (91.7)	52 (91.2)
Yes	14 (8.9)	3 (10.7)	6 (8.3)	5 (8.8)
* P*-value^(1)^				
C1		–	0.7073	1.0000
C2		0.7073	–	1.0000
C3		1.0000	1.0000	–
Localization				
N	14	3	6	5
Axilla, bones, breast and liver	1 (7.1)	0 (0)	1 (16.7)	0 (0)
Axillary and laterocervical nodes, liver	1 (7.1)	0 (0)	1 (16.7)	0 (0)
Lymph nodes and meninges	1 (7.1)	1 (33.3)	0 (0)	0 (0)
Liver and bone	1 (7.1)	0 (0)	1 (16.7)	0 (0)
Bone	2 (14.3)	0 (0)	0 (0)	2 (40.0)
Bone, lymph node, lung	1 (7.1)	0 (0)	0 (0)	1 (20.0)
Bones	1 (7.1)	0 (0)	1 (16.7)	0 (0)
Thorax carcinomatous lymphangitis, liver, bone, and subcutaneous cellular tissue	1 (7.1)	0 (0)	1 (16.7)	0 (0)
Local	1 (7.1)	0 (0)	0 (0)	1 (20.0)
Breast	2 (14.3)	1 (33.3)	0 (0)	1 (20.0)
Skin	1 (7.1)	1 (33.3)	0 (0)	0 (0)
Lung	1 (7.1)	0 (0)	1 (16.7)	0 (0)
* P*-value^(2)^				
C1		–	0.3423	0.3194
C2		0.3423	–	0.2757
C3		0.3194	0.2757	–
Exitus				
N	156	28	72	56
No	149 (95.5)	27 (96.4)	67 (93.1)	55 (98.2)
Yes	7 (4.5)	1 (3.6)	5 (6.9)	1 (1.8)
* P*-value^(1)^				
C1		–	1.0000	1.000
C2		1.0000	–	0.2300
C3		1.0000	0.2300	–

*C1* center 1, *C2* center 2, *C3* center 3

^(1)^Fisher’s exact test comparing each center with the other two

^(2)^Pearson chi-squared test comparing each center with the other two

**Table 4:** Surgery characteristics according to recurrence

	No recurrence (*N* = 143)	Recurrence (*N* = 14)	*P *value
TTL (copies/μL)			
Mean (SD)	26,419 (142,531)	19,301 (55,533)	0.0367^(1)^
TTL (copies/μL), *n* (%)			
≤ 250	107 (74.8)	7 (50.0)	0.0274^(2)^
> 250 a ≤ 5000	20 (14.0)	3 (21.4)	
> 5000 a ≤ 15,000	6 (4.2)	1 (7.1)	
> 15,000	10 (7.0)	3 (21.4)	
OSNA, *n* (%)			
No	0 (0)	2 (14.3)	0.0074^(3)^
Yes	143 (100.0)	12 (85.7)	
ALND, *n* (%)			
No	126 (88.1)	10 (71.4)	0.0966^(3)^
Yes	17 (11.9)	4 (28.6)	

*SD* standard deviation, *OSNA* one step nucleic acid amplification, *ALND* axillary lymph node dissection

^(1)^Mann–Whitney test

^(2)^Linear-by-linear association test

^(3)^Fisher’s exact test

**Fig. 1:**
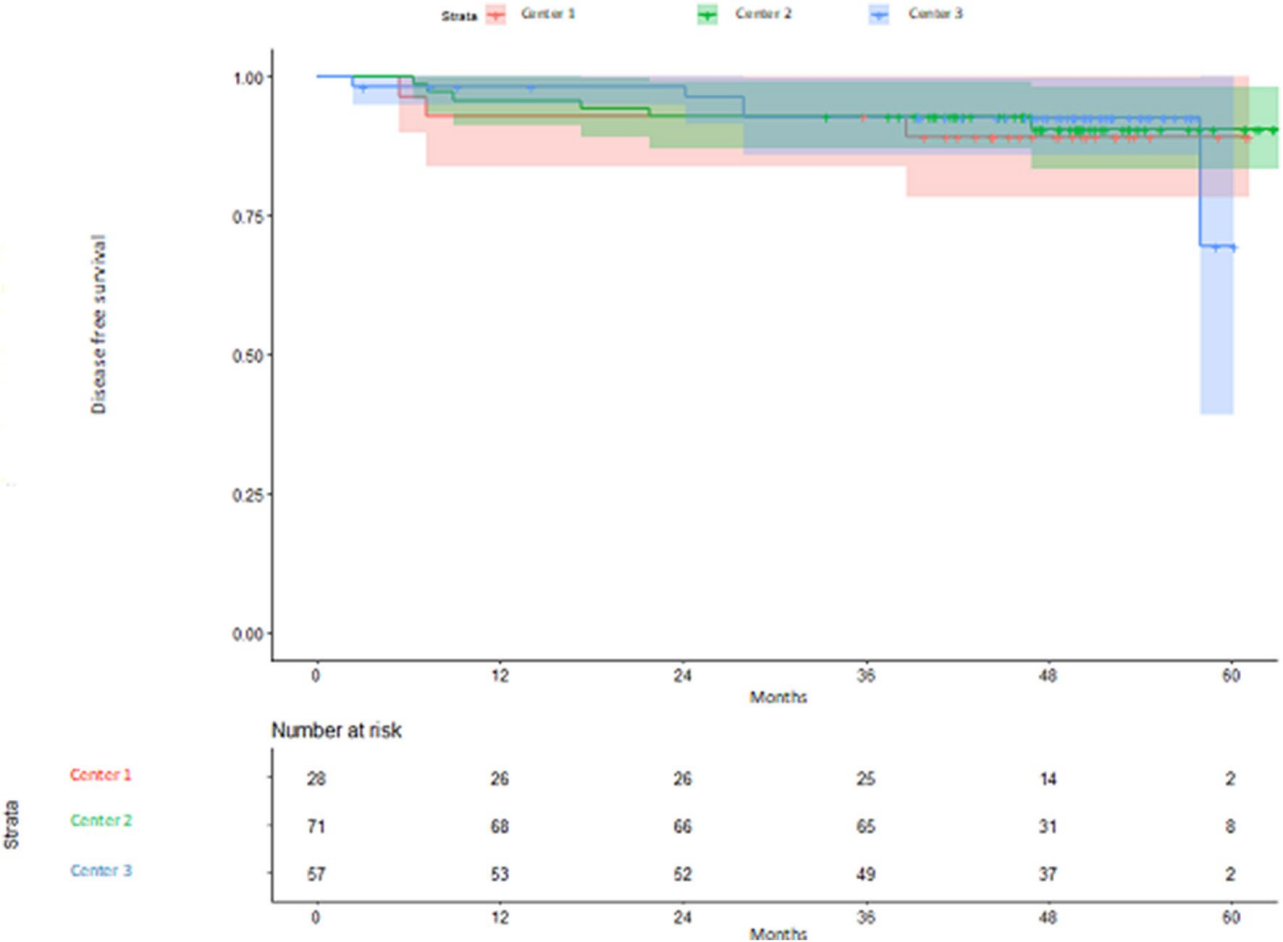
Kaplan–Meier estimates of disease-free survival according to center

Regarding DFS (main objective), no significant differences were observed between centers, with different TTL cut-off points per center (HR center 2 vs 1: 0.77; 95% confidence interval [95% CI] 0.19–3.07, HR center 3 vs 1: 0.83 95% CI 0.20–3.48; *p* = 0.707 and 0.799 respectively) (Fig. 1).

**Fig. 2:**
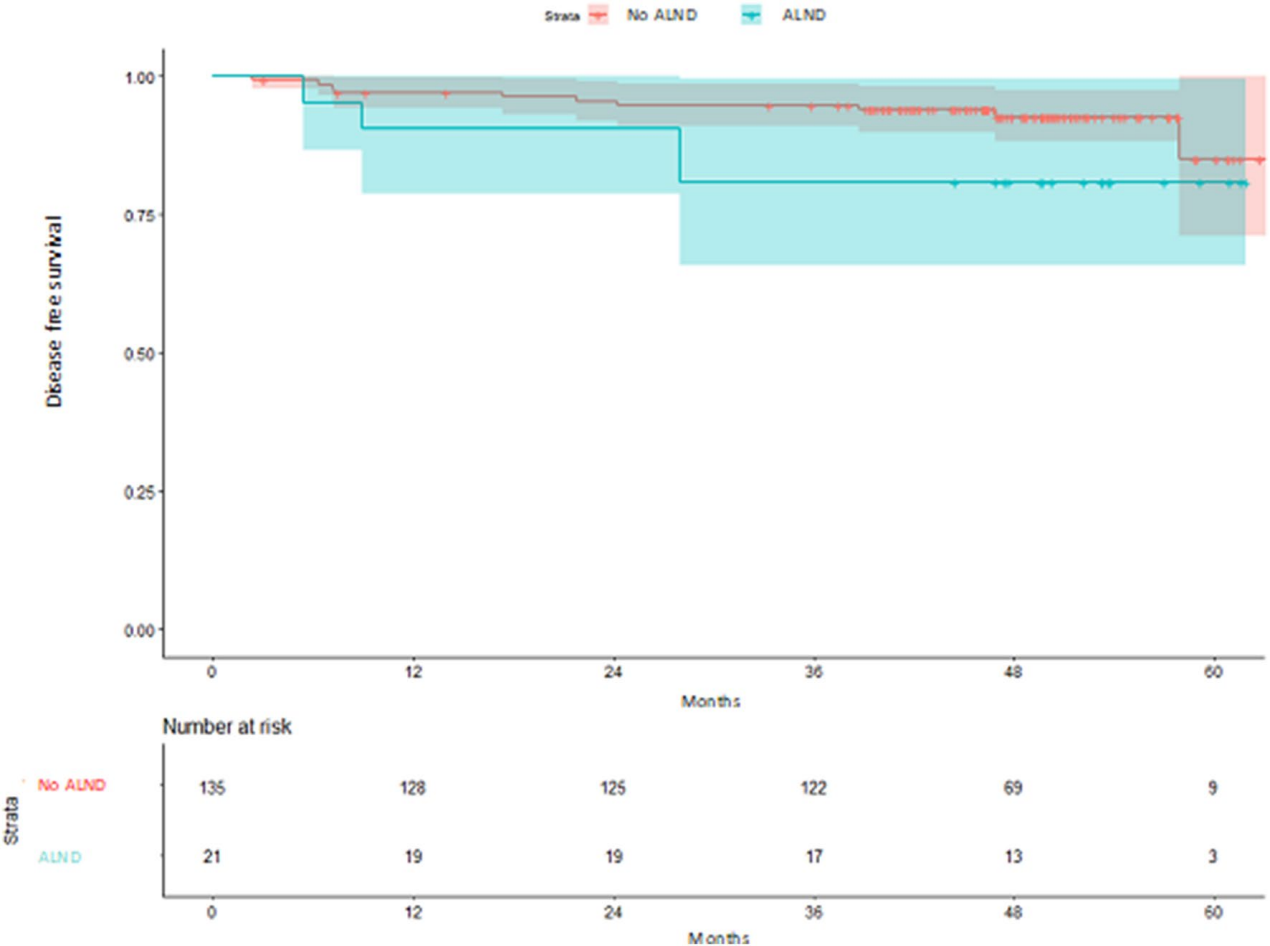
Kaplan–Meier estimates of disease-free survival according to ALND

**Fig. 3:**
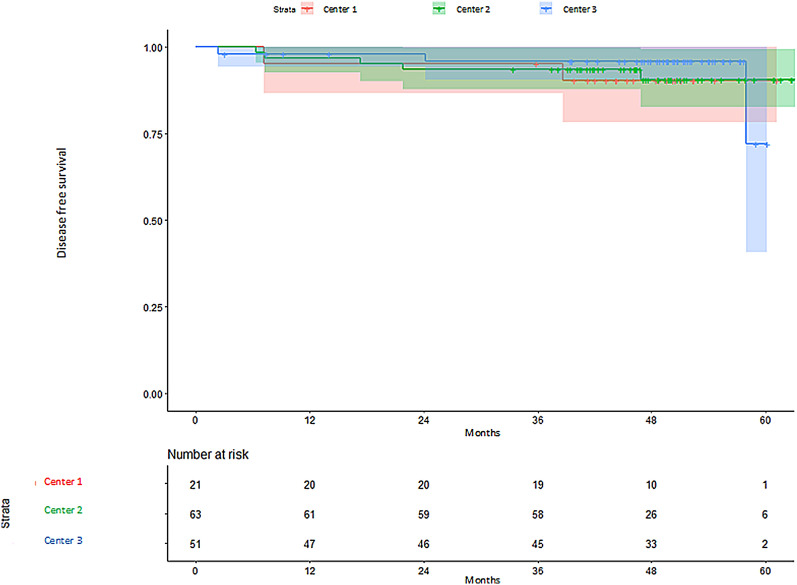
Kaplan–Meier estimates of disease-free survival in patients without ALND according to center

**Fig. 4:**
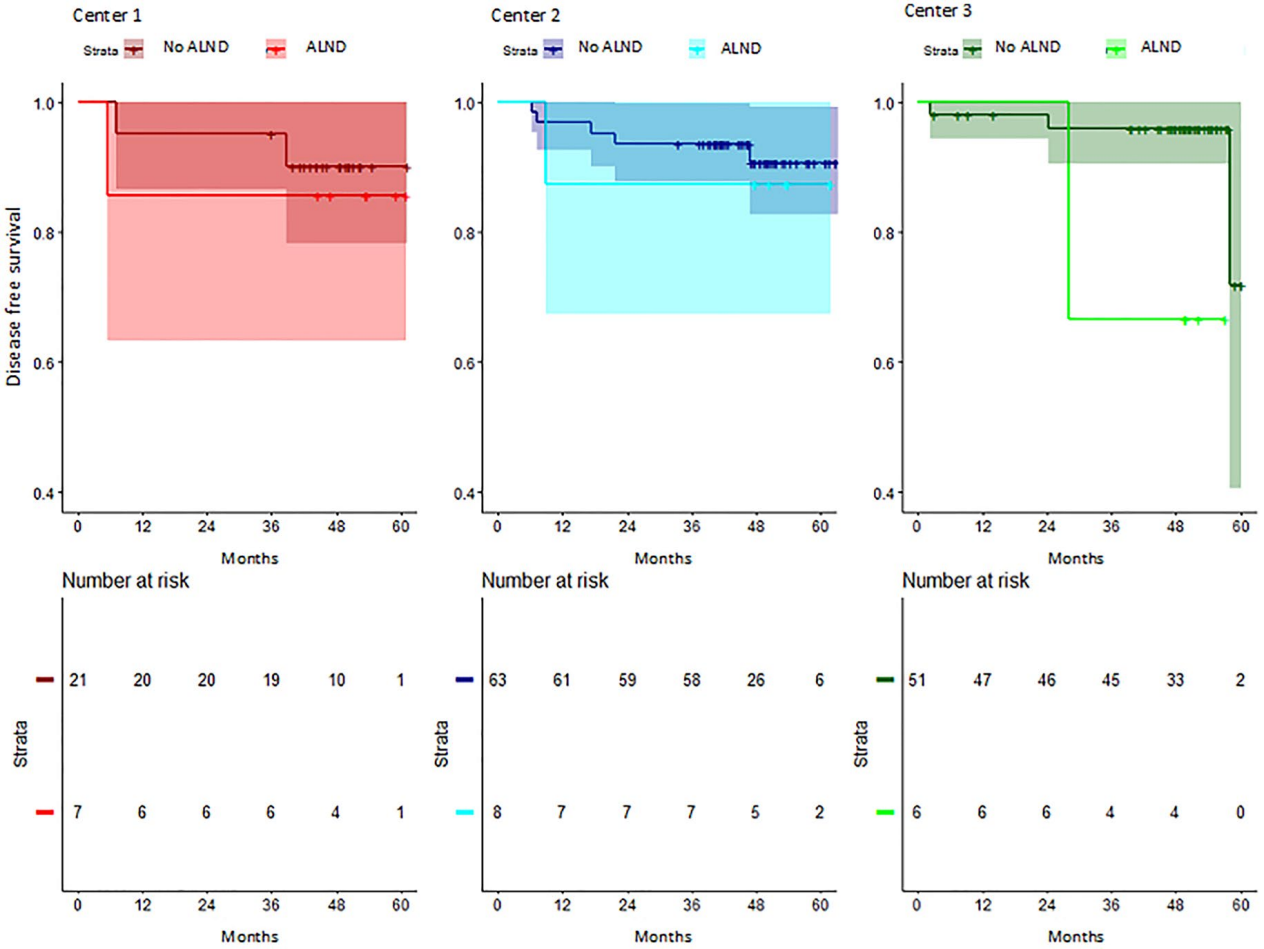
Kaplan–Meier estimates of disease-free survival according to center and ALND

Regarding DFS according to ALND, patients with ALND had a shorter DFS, although differences were not statistically significant (HR 2.43, 95% CI 0.76–7.79; *p* = 0.136) (Fig. 2). In those patients with no ALND performed, no significant differences were observed between centers (HR center 2 vs 1: 0.77, 95% CI 0.77–4.00 *p* = 0.758; HR center 3 vs 1: 0.58, 95% CI 0.58–3.50 *p* = 0.555) (Fig. 3). Analysis of DFS stratified by center according to ALND, showed no differences between centers (HR center 2 vs 1: 0.76, 95% CI 0.13–4.35* p* = 0.756; HR center 3 vs 1: 0.69, 95% CI 0.10–4.80 *p* = 0.706) (Fig. 4). Patients without ALND were insufficient to establish a relationship between DFS and center. Within these patients, less than 10% had recurrence, with no significant differences between centers (Table S5).

**Fig. 5:**
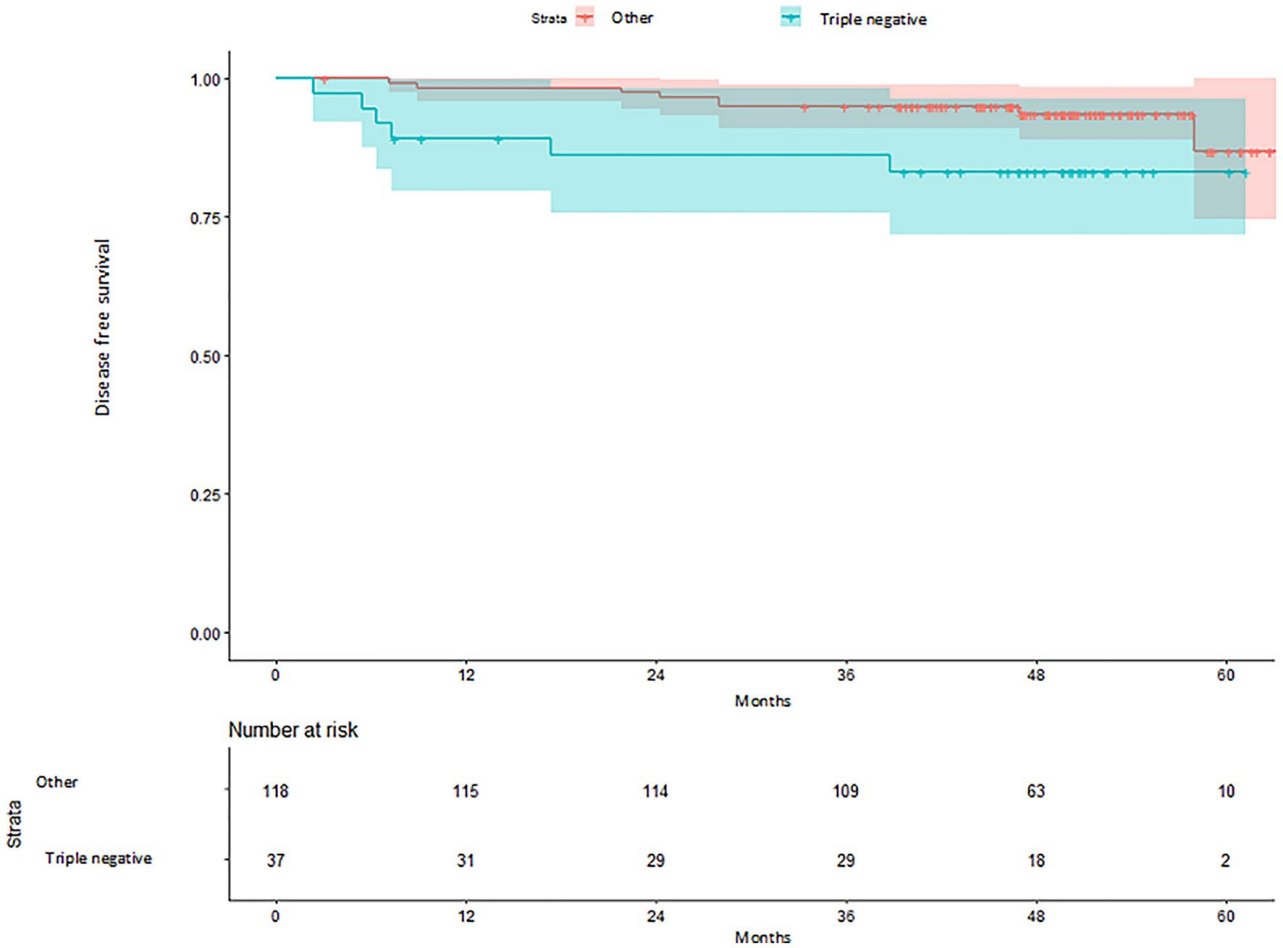
Kaplan–Meier estimates of disease-free survival according to molecular-like subtype

The evaluation of the secondary objective of DFS according to the molecular subtype of BC showed that patients with a triple negative subtype have a worse prognosis than those with other molecular subtypes (HR 2.82; 95% CI 0.97–8.16; *p* = 0.056) (Fig. 5).

## Discussion

A consensus about axillary approach in patients with BC after neoadjuvant therapy, especially in early clinical stages, is still missing. The SNB has been accepted as a good tool for stablishing the axillary status, with the aim to avoid unnecessary ALND, but SLN histological assay is limited due to tissue alterations resulting from the previous systemic treatment. OSNA analysis may avoid these tissue limitations and provide a quantitative result.

Although previous studies have demonstrated the value of TTL as a prognostic marker for non-sentinel node disease in patients with BC with or without NAST, the cut-off to indicate ALND differs significantly among groups, especially after NAST [16, 17].

This retrospective study compared the outcomes (i.e., DFS) of BC patients who, after NAST, underwent BC surgery with intraoperative SLNB and analysis with the OSNA technique in three centers, with different ALND indication criteria based on TTL values. No significant differences in DFS were observed between centers performing ALND at TTL > 250, > 5000, and > 15,000 CK19 mRNA copies/μL.

The OSNA method quantifies the number of CK 19 mRNA copies/μL, classifying SLNBs with TTL < 250 copies/μL as negative, with 250–5000 copies/μL as micrometastasis, and with > 5000 copies/μL as macrometastasis [22]. In this study, patients underwent ALND following the criteria of each participating center. According to the definitions of the OSNA method and center criteria, all patients with micrometastases underwent ALND in center 1, while only those with macrometastases underwent ALND in centers 2 and 3, with different cut-off between both centers: > 5000 copies/μL in center 2 and > 15,000 copies/μL in center 3. The decision of the study centers to perform ALND according to TTL and not number of affected SLNs is in line with recent reports describing TTL in SLNs as an independent predictor of axillary involvement and showing a higher predictive ability of SLN involvement compared to the number of affected SLNs [14, 20, 23–27].

Few studies have provided prognostic information about the relationships between TTL in SLNs and prognosis (i.e., DFS) in patients with BC who have received prior NAST. NEOVATTL study was the first published study that assessed the predictive and prognostic value of TTL derived from molecular analysis of the SLN in BC patients after neoadjuvant systemic therapy. This study demonstrated that a TTL > 15,000 copies/μL predicted non-sentinel axillary affectation and reported that TTL > 25,000 copies/μL was associated with a higher risk of disease recurrence in 316 patients with a mean TTL of (42,314 ± 248,208) [17]. However, no significant difference in DFS prognosis between patients with a TTL of < 250 copies/μL versus ≥ 250–25,000 copies/μL was observed. With these findings, Vieites et al. suggested that small metastases (> 250 and ≤ 25,000 copies/μL) detected by OSNA have similar prognostic value to negative nodes, with clinical outcomes no worse than a patient with negative nodes. In this study evaluating 157 patients, the results in terms of DFS were similar between centers with TTL cut-off values for ALND of > 250, > 5000, and > 15,000 CK19 mRNA copies/μL. Based on these results, axillary management could be conservative in patients with TTL lower than 15,000 copies/μL, avoiding unnecessary ALND. Considering the previously identified TTL cut-off of 25,000 copies/μL to predict disease recurrence, it is possible that even more conservative approaches (i.e., performing ALND at > 15,000 and < 25,000 copies/μL) may have resulted in similar outcomes.

Regarding secondary objectives, concerning to the relationship between BC subtype and DFS, the results of this study have demonstrated that patients with a triple negative subtype had a worse prognosis in terms of DFS than those with other BC molecular subtypes. These findings are consistent with current scientific evidence describing a relationship between triple-negative BC and lower survival compared to the other BC subtypes [27–30].

Compared to the NEOVATTL study, the distribution of the percentage of patients with recurrences within the different cut-off groups was similar. Thus, in the NEOVATTL study, disease recurrence rates were 51.3% in the group of patients with TTL of < 250 copies/μL; 23.1% in those with TTL between 250 and 25,000 copies/μL, and 25.6% in the patients with > 25,000 copies/μL, with an overall disease recurrence rate of 12.4% (39 patients) [17]. In this study, also half of the patients with BC recurrence had TTL < 250 copies/μL with an overall disease recurrence rate of 8.9% (14 patients). Those results suggest that other clinicopathological factors, such as the surrogate molecular subtype, may influence the risk of recurrence.

Although ALND has been associated with significant morbidity (including the risk of developing lymphedema of the upper limb, paresthesia, pain, and restriction of motion of the shoulder girdle), the surgical strategy to the axillary approach, depending on the results of the SLN study, continues to vary widely in clinical practice. Thus, while some follow the criterion proposed by the ACOSOG-Z0011 Trial, according to which ALND could be avoided in patients with T1-2 BC and one or two positive SLNs, others use the value of the TTL detected in the SLN(s) after analysis with OSNA [31]. Furthermore, according to some authors, this method adds accuracy to the node assay after NAST because it measures CK19 derived from viable tumor cells, providing accurate information about residual tumor cells [21]. In this second case, even though the OSNA method is a standardized technique, there is no agreement between the groups on the cut-off point from which ALND should be performed. In this regard, the main objective of this study was to compare prognosis among three populations of BC patients after NAST with different surgical approaches according to TTL levels and no significant differences in DFS were found. These findings suggest that restricting ALND to those patients with TTL > 15,000 CK mRNA copies/μL is a reliable approximation avoiding unnecessary morbidities caused by ALND.

The limitations of this study are attributable to the retrospective nature of its design, including the risk of patient selection bias. In addition to the limited number of patients in this study and the presence of variability in patient follow-up times, the low number of patients with ALND (*n* = 21) and the few recorded events may have influenced the analysis of DFS according to ALND. Finally, the uneven recruitment between centers resulted in an uneven distribution of patients between groups.

Despite these limitations, the results of this study, showing similar outcomes irrespective of the axillary approach based on different TTL cut-off values, could guide decision-making regarding ALND and breast-conserving surgery in BC patients who have received NAST. Prospective and multicenter randomized controlled studies are necessary to determine the predictive value of TTL for the diagnosis of non-SLN metastases and establish cut-off points that could guide the surgical strategy in terms of the axillary approach after NAST in patients with BC.

## Conclusion

In this study, no significant differences in DFS were observed between three centers with different surgical approaches to ALND based on different TTL cut-offs obtained in the SNs assay, in patients with BC after NAST. These results suggest that restricting ALND to those patients with TTL ≥ 15,000 CK mRNA copies/μl is a reliable approximation avoiding unnecessary morbidities caused by ALND.

## Supplementary Information

Below is the link to the electronic supplementary material.

Supplementary file1 (DOCX 39 KB)

## Data Availability

The datasets generated during and/or analyzed during the current study are not publicly available but are available from the corresponding author on reasonable request.

## References

[1] Bray F, Ferlay J, Soerjomataram I (2018). Global cancer statistics 2018: GLOBOCAN estimates of incidence and mortality worldwide for 36 cancers in 185 countries. CA Cancer J Clin.

[2] Sociedad Española de Oncología Médica (SEOM) LAS CIFRAS DEL CANCER EN ESPAÑA 2022. https://seom.org/images/LAS_CIFRAS_DEL_CANCER_EN_ESPANA_2022.pdf. Accessed 14 Oct 2022

[3] Li X, Wang M, Wang M (2019). Predictive and prognostic roles of pathological indicators for patients with breast cancer on neoadjuvant chemotherapy. J Breast Cancer.

[4] Popat S, Smith IE (2005). Re: neoadjuvant versus adjuvant systemic treatment in breast cancer: a meta-analysis. J Natl Cancer Inst.

[5] Kaufmann M, von Minckwitz G, Mamounas EP (2012). Recommendations from an international consensus conference on the current status and future of neoadjuvant systemic therapy in primary breast cancer. Ann Surg Oncol.

[6] Mamounas EP (2018). Optimizing surgical management of the axilla after neoadjuvant chemotherapy: an evolving story. Ann Surg Oncol.

[7] Navarro-Cecilia J, Dueñas-Rodríguez B, Luque-López C (2013). Intraoperative sentinel node biopsy by one-step nucleic acid amplification (OSNA) avoids axillary lymphadenectomy in women with breast cancer treated with neoadjuvant chemotherapy. Eur J Surg Oncol (EJSO).

[8] Loibl S, von Minckwitz G, Raab G (2006). Surgical procedures after neoadjuvant chemotherapy in operable breast cancer: results of the GEPARDUO trial. Ann Surg Oncol.

[9] Pecha V, Kolarik D, Kozevnikova R (2011). Sentinel lymph node biopsy in breast cancer patients treated with neoadjuvant chemotherapy. Cancer.

[10] Vieites B, López-García MÁ, Castilla C (2016). CK19 expression in breast tumours and lymph node metastasis after neoadjuvant therapy. Histopathology.

[11] Chu PG, Weiss LM (2002). Keratin expression in human tissues and neoplasms. Histopathology.

[12] Espinosa-Bravo M, Navarro-Cecilia J, Ramos Boyero M (2017). Intraoperative assessment of sentinel lymph node by one-step nucleic acid amplification in breast cancer patients after neoadjuvant treatment reduces the need for a second surgery for axillary lymph node dissection. Breast.

[13] Takamoto K, Shimazu K, Naoi Y (2016). One-step nucleic acid amplification assay for detection of axillary lymph node metastases in breast cancer patients treated with neoadjuvant chemotherapy. Ann Surg Oncol.

[14] Espinosa-Bravo M, Sansano I, Pérez-Hoyos S (2013). Prediction of non-sentinel lymph node metastasis in early breast cancer by assessing total tumoral load in the sentinel lymph node by molecular assay. Eur J Surg Oncol (EJSO).

[15] Peg V, Espinosa-Bravo M, Vieites B (2013). Intraoperative molecular analysis of total tumor load in sentinel lymph node: a new predictor of axillary status in early breast cancer patients. Breast Cancer Res Treat.

[16] Peg V, Sansano I, Vieites B (2017). Role of total tumour load of sentinel lymph node on survival in early breast cancer patients. The Breast.

[17] Vieites B, López-García MÁ, Martín-Salvago MD (2021). Predictive and prognostic value of total tumor load in sentinel lymph nodes in breast cancer patients after neoadjuvant treatment using one-step nucleic acid amplification: the NEOVATTL study. Clin Transl Oncol.

[18] Rebollo-Aguirre ÁC, Gallego-Peinado M, Sánchez-Sánchez R (2013). Biopsia del ganglio centinela después de la quimioterapia neoadyuvante en pacientes con cáncer de mama operable y ganglios axilares positivos al diagnóstico. Rev Esp Med Nucl Imagen Mol.

[19] Rubio IT, Espinosa-Bravo M, Rodrigo M (2014). Nomogram including the total tumoral load in the sentinel nodes assessed by one-step nucleic acid amplification as a new factor for predicting nonsentinel lymph node metastasis in breast cancer patients. Breast Cancer Res Treat.

[20] Hunter-Smith AE, Rayter Z (2018). One-step nucleic acid amplification: the possible value in assessing sentinel lymph node metastasis during mastectomy. Breast Cancer.

[21] Tsujimoto M, Nakabayashi K, Yoshidome K (2007). One-step nucleic acid amplification for intraoperative detection of lymph node metastasis in breast cancer patients. Clin Cancer Res.

[22] Laohawiriyakamol S, Mahattanobon S, Puttawibul P (2022). Intraoperative molecular analysis of total tumor load in sentinel lymph node: a predictor of axillary status in early breast cancer. Asian Pacific J Cancer Prevent.

[23] Piñero A, Canteras M, Moreno A (2013). Multicenter validation of two nomograms to predict non-sentinel node involvement in breast cancer. Clin Transl Oncol.

[24] di Filippo F, Giannarelli D, Bouteille C (2015). Elaboration of a nomogram to predict non sentinel node status in breast cancer patients with positive sentinel node, intra-operatively assessed with one step nucleic acid amplification method. J Exp Clin Cancer Res.

[25] Ohi Y, Umekita Y, Sagara Y (2012). Whole sentinel lymph node analysis by a molecular assay predicts axillary node status in breast cancer. Br J Cancer.

[26] Teramoto A, Shimazu K, Naoi Y (2014). One-step nucleic acid amplification assay for intraoperative prediction of non-sentinel lymph node metastasis in breast cancer patients with sentinel lymph node metastasis. The Breast.

[27] Foulkes WD, Smith IE, Reis-Filho JS (2010). Triple-negative breast cancer. N Engl J Med.

[28] Vargo JA, Beriwal S, Ahrendt GM (2011). Molecular class as a predictor of locoregional and distant recurrence in the neoadjuvant setting for breast cancer. Oncology.

[29] Li X, Yang J, Peng L (2017). Triple-negative breast cancer has worse overall survival and cause-specific survival than non-triple-negative breast cancer. Breast Cancer Res Treat.

[30] Gonçalves H, Guerra MR, Duarte Cintra JR (2018). Survival study of triple-negative and non-triple-negative breast cancer in a Brazilian cohort. Clin Med Insights Oncol.

[31] Giuliano AE, Ballman K, McCall L (2017). Effect of axillary dissection vs no axillary dissection on 10-year overall survival among women with invasive breast cancer and sentinel node metastasis. JAMA.

