# Supplementary material for: Value of total tumor load as a clinical and pathological factor in the prognosis of breast cancer patients receiving neoadjuvant treatment. Comparison of three populations with three different surgical approaches: NEOVATTL Pro 3 Study
Source: Breast Cancer Res Treat. 2023 May 23;200(2):203–15. doi: 10.1007/s10549-023-06954-8 (PMC10241680; doi:10.1007/s10549-023-06954-8)
Supplement: Supplementary file 1 — Supplementary file1 (DOCX 39 KB) [file 10549_2023_6954_MOESM1_ESM.docx]

# Supplementary Tables

| **Table S1.** Hormonal receptors and molecular- like subtype according to center, n (%) | | | | |
| --- | --- | --- | --- | --- |
|  | **Total** | **C1** | **C2** | **C3** |
| **ER** | | | | |
| N | 157 | 28 | 72 | 57 |
| 0 | 53 (33.8) | 13 (46.4) | 16 (22.2) | 24 (42.1) |
| 0-10 | 7 (4.5) | 1 (3.6) | 3 (4.2) | 3 (5.3) |
| 10-20 | 1 (0.6) | 0 (0) | 0 (0) | 1 (1.8) |
| 30-60 | 7 (4.5) | 2 (7.1) | 3 (4.2) | 2 (3.5) |
| 60-90 | 29 (18.5) | 6 (21.4) | 17 (23.6) | 6 (10.5) |
| 90-100 | 60 (38.2) | 6 (21.4) | 33 (45.8) | 21 (36.8) |
| *P*-value^(1)^ |  |  |  |  |
| C1 |  | - | 0.0088 | 0.5974 |
| C2 |  | 0.0088 | - | 0.0156 |
| C3 |  | 0.5974 | 0.0156 | - |
| **PR** | | | | |
| N | 157 | 28 | 72 | 57 |
| 0 | 75 (47.8) | 17 (60.7) | 26 (36.1) | 32 (56.1) |
| 0-10 | 15 (9.6) | 4 (14.3) | 8 (11.1) | 3 (5.3) |
| 10-20 | 6 (3.8) | 0 (0) | 5 (6.9) | 1 (1.8) |
| 20-30 | 5 (3.2) | 1 (3.6) | 2 (2.8) | 2 (3.5) |
| 30-60 | 15 (9.6) | 1 (3.6) | 10 (13.9) | 4 (7.0) |
| 60-90 | 18 (11.5) | 4 (14.3) | 9 (12.5) | 5 (8.8) |
| 90-100 | 23 (14.6) | 1 (3.6) | 12 (16.7) | 10 (17.5) |
| *P*-value^(1)^ |  |  |  |  |
| C1 |  | - | 0.0235 | 0.2424 |
| C2 |  | 0.0235 | - | 0.2072 |
| C3 |  | 0.2424 | 0.2072 | - |
| **HER2** | | | | |
| N | 157 | 28 | 72 | 57 |
| 0 | 34 (21.7) | 9 (32.1) | 9 (12.5) | 16 (28.1) |
| 1 | 25 (15.9) | 3 (10.7) | 17 (23.6) | 5 (8.8) |
| 2+ ISH (-) | 41 (26.1) | 3 (10.7) | 28 (38.9) | 10 (17.5) |
| 2+ ISH (+) | 9 (5.7) | 2 (7.1) | 7 (9.7) | 0 (0) |
| 3 | 16 (10.2) | 0 (0) | 0 (0) | 16 (28.1) |
| 3+ | 32 (20.4) | 11 (39.3) | 11 (15.3) | 10 (17.5) |
| *P*-value^(1)^ |  |  |  |  |
| C1 |  | - | 0.5358 | 0.895 |
| C2 |  | 0.5358 | - | 0.2238 |
| C3 |  | 0.8956 | 0.2238 | - |
| **Ki67 (% of positive cells)** | | | | |
| N | 157 | 28 | 72 | 57 |
| <10 | 11 (7.0) | 1 (3.6) | 6 (8.3) | 4 (7.0) |
| 10-20 | 25 (15.9) | 0 (0) | 16 (22.2) | 9 (15.8) |
| 20-30 | 41 (26.1) | 9 (32.1) | 20 (27.8) | 12 (21.1) |
| 30-60 | 51 (32.5) | 8 (28.6) | 20 (27.8) | 23 (40.4) |
| 60-90 | 27 (17.2) | 10 (35.7) | 10 (13.9) | 7 (12.3) |
| 90-100 | 2 (1.3) | 0 (0) | 0 (0) | 2 (3.5) |
| *P*-value^(1)^ |  |  |  |  |
| C1 |  | - | 0.003 | 0.0791 |
| C2 |  | 0.0037 | - | 0.1719 |
| C3 |  | 0.079 | 0.1719 | - |
| **Molecular-like subtype** | | | | |
| N | 156 | 28 | 72 | 56 |
| HER2 | 16 (10.3) | 3 (10.7) | 3 (4.2) | 10 (17.9) |
| LUM A | 28 (17.9) | 0 (0) | 18 (25.0) | 10 (17.9) |
| LUM B | 34 (21.8) | 4 (14.3) | 23 (31.9) | 7 (12.5) |
| LUM B-HER2 | 41 (26.3) | 10 (35.7) | 15 (20.8) | 16 (28.6) |
| TN | 37 (23.7) | 11 (39.3) | 13 (18.1) | 13 (23.2) |
| *P*-value^(2)^ |  |  |  |  |
| C1 |  | - | 0.0026 | 0.1050 |
| C2 |  | 0.0026 | - | 0.0122 |
| C3 |  | 0.1050 | 0.0122 | - |

C1, center 1; C2, center 2; C3, center 3, ER, estrogen receptor; PR, progesterone receptor; HER2, human epidermal growth factor receptor 2; ISH, in situ hybridization; LUM A, luminal A; LUM B, luminal B.

^(1)^ Linear-by-linear association test comparing each center with the other two; ^(2)^ Pearson chi-squared test comparing each center with the other two.

| **Table S2.** Neoadjuvant characteristics by center, n (%) | | | | |
| --- | --- | --- | --- | --- |
|  | **Total** | **C1** | **C2** | **C3** |
| **CNB lymph node** | | | | |
| N | 157 | 28 | 72 | 57 |
| No | 108 (68.8) | 15 (53.6) | 58 (80.6) | 35 (61.4) |
| Yes, (-) | 24 (15.3) | 7 (25.0) | 10 (13.9) | 7 (12.3) |
| Yes, (+) | 25 (15.9) | 6 (21.4) | 4 (5.6) | 15 (26.3) |
| *P*-value^(1)^ |  |  |  |  |
| C1 |  | - | 0.0139(1) | 0.3293 |
| C2 |  | 0.0139(1) | - | 0.0041 |
| C3 |  | 0.3293(1) | 0.0041(1) | - |
| **Neoadjuvant treatment** | | | | |
| N | 157 | 28 | 72 | 57 |
| ANT+TAX | 81 (51.6) | 9 (32.1) | 46 (63.9) | 26 (45.6) |
| ANT+TAX+HER2 | 57 (36.3) | 13 (46.4) | 20 (27.8) | 24 (42.1) |
| OTHER | 19 (12.1) | 6 (21.4) | 6 (8.3) | 7 (12.3) |
| *P*-value^(1)^ |  |  |  |  |
| C1 |  | - | 0.0129(1) | 0.3799 |
| C2 |  | 0.0129(1) | - | 0.1159 |
| C3 |  | 0.3799(1) | 0.1159(1) | - |
| **Breast Radiological Response** | | | | |
| N | 155 | 28 | 72 | 55 |
| No | 81 (51.6) | 1 (3.6) | 0 (0) | 3 (5.5) |
| Partial | 57 (36.3) | 18 (64.3) | 57 (79.2) | 21 (38.2) |
| Total | 19 (12.1) | 9 (32.1) | 15 (20.8) | 31 (56.4) |
| *P*-value^(2)^ |  |  |  |  |
| C1 |  | - | 0.4362(2) | 0.1020 |
| C2 |  | 0.4362(2) | - | 0.0013 |
| C3 |  | 0.1020(2) | 0.0013(2) | - |
| **Axillar Radiological Response** | | | | |
| N | 127 | 28 | 72 | 27 |
| No | 4 (2.6) | 0 (0) | 63 (87.5) | 1 (3.7) |
| Partial | 96 (61.9) | 2 (7.1) | 4 (5.6) | 2 (7.4) |
| Total | 55 (35.5) | 26 (92.9) | 5 (6.9) | 24 (88.9) |
| *P*-value^(2)^ |  |  |  |  |
| C1 |  | - | < 0.0001 | 0.4406 |
| C2 |  | < 0.0001 | - | < 0.0001 |
| C3 |  | 0.440 | < 0.0001 | - |

C1, center 1; C2, center 2; C3, center 3; CNB, core needle biopsy; ANT, anthracyclines; TAX, taxanes; HER2, human epidermal growth factor receptor 2.

^(1)^ Pearson chi-squared test comparing each center with the other two; ^(2)^ Linear-by-linear association test comparing each center with the other two

| **Table S3.** TTL values according to molecular subtype (other vs. triple negative) | | | |
| --- | --- | --- | --- |
|  | **Others**  **(N=119)** | **Triple negative**  **(N=37)** | ***P*-value** |
| **TTL (copies/μL)** | | | |
| Mean (SD) | 33156 (156604) | 2763 (11612) | 0,6048^(1)^ |
| CI 95% | (5019; 61293) | (-979; 6504) |  |
| **Log10 TTL** | | | |
| Mean (SD) | 1,0 (1,8) | 0,8 (1,5) | 0,6048^(1)^ |
| CI 95% | (0,7; 1,4) | (0,3; 1,3) |  |
| **TTL (copies/μL), n (%)** | | | |
| ≤250 | 86 (72,3) | 28 (75,7) | 0,4378^(2)^ |
| > 250 to ≤ 5000 | 16 (13,4) | 6 (16,2) |  |
| > 5000 to ≤ 15000 | 6 (5,0) | 1 (2,7) |  |
| > 15000 | 11 (9,2) | 2 (5,4) |  |

^(1)^ Mann-Whitney test; ^(2)^ Linear-by-linear association test

**Table S4.** Unnecessary lymphadenectomy according to center 3 threshold.

| **Centre** | **TTL < 15000 and negative ALND** |
| --- | --- |
| 1 | 14.3% (1) |
| 2 | 25.0% (2) |
| 3 | 0.0% (0) |
| Total | 14.3% (3) |

| **Table S5.** Tumor recurrence according to center in patients with no ALND, n (%) | | | | |
| --- | --- | --- | --- | --- |
|  | **Total** | **C1** | **C2** | **C3** |
| **Patients with recurrence** | | | | |
| N | 21 | 64 | 51 | 21 |
| No | 19 (90.5) | 59 (92.2) | 48 (94.1) | 19 (90.5) |
| Yes | 2 (9.5) | 5 (7.8) | 3 (5.9) | 2 (9.5) |
| *P*-value^(1)^ |  |  |  |  |
| C1 |  | - | 1.0000 | 0.6249 |
| C2 |  | 1.0000 | - | 1.0000 |
| C3 |  | 0.6249 | 1.0000 | - |

^(1)^ Fisher’s exact test comparing each center with the other two
